# Synthetic Virus-Derived Nanosystems (SVNs) for Delivery and Precision Docking of Large Multifunctional DNA Circuitry in Mammalian Cells

**DOI:** 10.3390/pharmaceutics12080759

**Published:** 2020-08-11

**Authors:** Francesco Aulicino, Julien Capin, Imre Berger

**Affiliations:** 1 Bristol Synthetic Biology Centre BrisSynBio, School of Biochemistry, 1 Tankard’s Close, University of Bristol, Bristol BS8 1TD, UK; jc16969@bristol.ac.uk; 2 Max Planck Bristol Centre for Minimal Biology, School of Chemistry, Cantock’s Close, University of Bristol, Bristol BS8 1TS, UK

**Keywords:** baculovirus, CRISPR, gene editing, genome engineering, precision DNA docking

## Abstract

DNA delivery is at the forefront of current research efforts in gene therapy and synthetic biology. Viral vectors have traditionally dominated the field; however, nonviral delivery systems are increasingly gaining traction. Baculoviruses are arthropod-specific viruses that can be easily engineered and repurposed to accommodate and deliver large sequences of exogenous DNA into mammalian cells, tissues, or ultimately organisms. These synthetic virus-derived nanosystems (SVNs) are safe, readily customized, and can be manufactured at scale. By implementing clustered regularly interspaced palindromic repeats (CRISPR) associated protein (CRISPR/Cas) modalities into this system, we developed SVNs capable of inserting complex DNAs into genomes, at base pair precision. We anticipate a major role for SVNs as an attractive alternative to viral vectors in accelerating genome engineering and gene therapy applications in the future.

## 1. Introduction

Programmable DNA nucleases such as clustered regularly interspaced palindromic repeats (CRISPR) associated protein 9 (CRISPR/Cas9) have enormously contributed to rejuvenate and democratize the gene editing and gene therapy fields thanks to their efficiency, cost-effectiveness, and specificity, in a variety of model organisms in ex vivo and in vivo. Although CRISPR system was originally discovered in bacteria as a previously unknown adaptive immune response pathway, it was successfully repurposed into a gene editing tool when Cas9, an RNA guided DNA programmable nuclease, was shown to efficiently work in mammalian cells to produce double stranded DNA breaks (DSBs) at desired genomic loci with very high efficiency and little off-target effects [1,2].

According to the subsequent DNA repair mechanism, Cas9-induced DSBs can be resolved with the production of small insertion/deletions (indels) through the non-homologous end joining (NHEJ) DNA repair pathway, or precise gene editing outcomes when the homology-directed repair (HDR) pathway is exploited in combination with a suitable DNA template [2].

Remarkably, the applications of CRISPR technology extends beyond gene editing and catalytically inactive Cas9 has been successfully used, in fusion with various cofactors, to modulate gene expression [3], visualize single genomic loci in living cells [4], and change the epigenetic state of a given chromatin region [5].

As the first clinical trials involving CRISPR are being undertaken, significant effort is invested to fully transform this powerful gene editing tool into a reliable technology for next generation gene therapy interventions ranging from immunization and cancer therapy to gene replacement in vivo. More efficient Cas9 variants with fewer off-target effects have been engineered, and the extensive search for Cas variants and Cas-like proteins has led to the discovery of additional endonucleases which further expand the repertoire of targetable DNA sequences [6,7,8].

While CRISPR-mediated gene editing is highly efficient in “easy to transfect” cell lines, its efficiency drops sharply when switching to transfection recalcitrant cell lines, exacerbated when a DNA template has to be co-transfected in addition to guide RNA and Cas9 to achieve homology-directed repair. Delivering Cas9, sgRNA, and donor DNA simultaneously within the same cell and with the appropriate stoichiometric ratio remains a formidable challenge, often resulting in no editing events or the production of indels. Coupled with the cell cycle dependency of the HDR pathway and its context dependent activity [9], delivery efficiency represents an imposing roadblock to the development of future highly efficient CRISPR-based gene therapy interventions.

Nonviral delivery systems (e.g., electroporation, polymer-based, or lipid-based delivery of RNA, DNA, and proteins) can achieve remarkable editing efficiencies in cultured cells [10], however, at the expense of time-consuming parameter adaptation and at times toxic side effects at the cellular level [10]. Promisingly, lipid-based delivery of Cas9/sgRNA ribonucleoprotein (RNP) complexes can be harnessed in vivo to achieve gene knockouts, albeit with lower editing efficiency than in cultured cells [11,12,13]. Common bottlenecks to consider for nonviral delivery systems are scalability and manufacturing costs [14]. These technologies, although extremely safe, would likely continue to be confined to ex vivo gene editing, for which parameters such as cell confluency and culturing methods could be adjusted in a more straight-forward manner to achieve the highest delivery and gene editing efficacies. Furthermore, to the best of our knowledge, in vivo lipid-based delivery of RNPs has not been demonstrated to date for precise gene editing approaches in which concomitant delivery of a DNA donor is required.

In marked contrast, currently available, both ex vivo and in vivo, viral delivery systems (e.g., lentivirus, adenovirus, or adeno-associated viruses) are characterized by high transduction efficiency. However, pre-existing immune response in humans, undesired integration at CRISPR-produced DSBs [15] or randomly in the genome, impede their future applications as systemic gene delivery vectors. In addition, viral vector applications are constrained by their limited cargo capacity which is typically derived from the defined and invariable geometry of their viral capsids, forcing researchers to adopt alternative approaches such as splitting the CRISPR machinery across several virions, combined with using smaller Cas variants [16], split-intein lentiviral vectors (LV) [17], or multiple adeno-associated vectors (AAVs) [18], adding an entire layer of new stochastic challenges to the desired gene editing outcome.

At the boundary between viral and nonviral CRISPR delivery systems, emerges a third highly attractive option which is the use of baculoviral delivery vectors engineered from the *Autographa californica* multiple nucleopolyhedrovirus (AcMNPV). Baculoviral vectors (BVs) are technically viruses in narrowly defined arthropod hosts which have the unique advantage that they do not replicate in mammalian cells but can be easily engineered to efficiently transduce them. In this context, they are replication and integration deficient (alike nonviral delivery systems), while preserving a high transduction rate (alike viral delivery systems) together with a cost-effective and scalable manufacturing process. Synthetic virus-derived nanosystems (SVNs), in this respect, combine the best of both delivery system approaches.

Importantly, the baculoviral genome is characterized by a very large heterologous DNA cargo capacity, far superior to LVs, adenovirus (AV), and AAVs, due to a flexible viral envelope which simply grows when additional heterologous DNA is inserted [15,16,17]. Thus, baculoviral vectors are uniquely suited to accommodate all components of a CRISPR machinery on a single vector [19,20], rendering them highly suitable for both ex vivo and in vivo delivery, taking advantage of customizable tropism and the lack of pre-existing immune response in humans [21]. In this review, we discuss baculoviral delivery systems and how current vector systems can be converted, through genome engineering and pseudotyping, into bona fide synthetic viral nanosystems (SVNs) to enable the deployment of CRISPR toolkits in next-generation gene editing and genome engineering applications.

## 2. MultiBac Baculovirus Engineering

**Figure 1:**
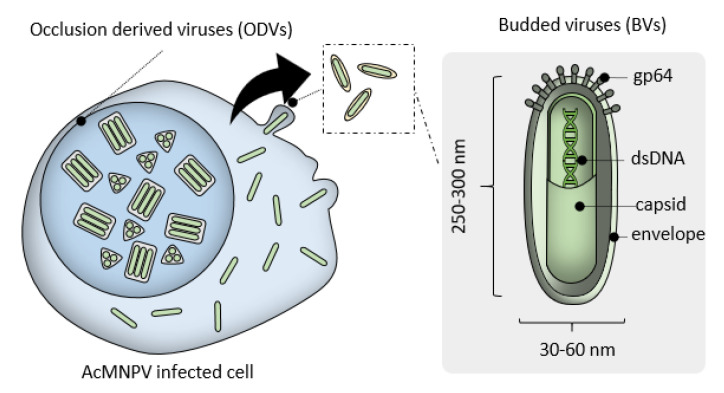
Wild-type *Autographa californica* multiple nucleopolyhedrovirus (AcMNP) produces two viral forms. Occlusion-derived viruses (ODVs) are found in occlusion bodies, embedded in protein matrixes mostly composed of polyhedrin protein. Budded viruses (BVs) are constantly released in the culturing media as rod-shaped particles surrounded by an envelope equipped with viral glycoprotein such as GP64, essential for BVs entry into target cells.

*Baculoviridae* are a family of enveloped dsDNA viruses with invertebrate host specificity. In nature, the genome of baculoviruses ranges between 80 and 180 kb. One of the most studied and translationally relevant members of the family is the *Autographa californica* multiple nucleopolyhedrovirus (AcMNPV) with a genome of ~135 kb encompassing 155 open reading frames (ORFs). The AcMNPV life cycle involves two viral forms that contain the same genetic information, which occupy distinct subcellular localization and serve different purposes. Budded viruses (BV) are enveloped virions which are constantly produced by infected cells, whereas occlusion-derived virions (ODVs) are highly structured viral forms organized in a matrix of polyhedrin proteins which shield the virions from adverse conditions such as cell death, desiccation, and thermolability, serving as an effective viral reservoir [22]. Although both viral forms play crucial roles during primary and secondary infections stages, ODVs are not required for viral propagation in laboratory culture and the polyhedrin protein can be removed from the AcMNPV genome. This leads to ablation of ODV production and does not affect budded virions, which are released directly into the culture media of AcMNPV infected cells (typically from *Spodoptera frugiperda*, *Trichoplusia ni* or *Bombyx mori*) (Figure 1).

This finding allowed for the generation of AcMNPV genomes in which the polyhedrin protein locus is substituted for an F replicon, a mini Tn7 attachment site within a LacZα gene, and a kanamycin resistance marker. The resulting 135 kb AcMNPV genome is propagated in bacteria as a large single copy plasmid called bacmid [23]. In the presence of a helper plasmid expressing the components of the Tn7 transposition machinery, a DNA insert can be shuttled into the bacmid into the Tn7 attachment site disrupting LacZα in blue/white screening. Then, the bacmid is isolated from *Escherichia coli* and introduced by transfection into insect cells to produce recombinant baculovirus expressing the protein of interest. The original system utilizing this approach, Bac-to-Bac (Invitrogen), has been extensively used for protein production in insect cells, usually coupled to strong viral promoters such as p10 or polh driving the expression of the protein of interest.

Alternatively, when these viral promoters are substituted for mammalian promoters (e.g., cytomegalovirus (CMV) promoter), the budded virions have also been shown to efficiently drive heterologous expression in mammalian cells, with little to no cytotoxic effect [21]. Importantly, baculoviral promoters are inactive in mammalian cells, rendering virus replication deficient and also integration deficient. Work involving baculoviruses is considered to be safe for users with the lowest biosafety level requirement (BSL-1) in most countries [19,21]. A variety of mammalian cells, including primary cell lines, have been efficiently transduced by baculovirus pseudotyped with glycoproteins from mammalian viruses, typically vesicular stomatitis virus (VSV) in a “BacMam” approach [24] with customized transfer plasmid, genetically encoded fluorescent probes, and cell-based assays available as off-the-shelf reagents.

The original BacMam approach mostly followed the one gene one vector paradigm. To fully take advantage of the superior cargo capacity of the baculoviral genome [20], diverse modalities of DNA assembly and insertion were developed and implemented in our laboratory giving rise to the MultiBac system, originally conceived to produce large multiprotein complexes with many subunits in insect cells [25]. We described the technical aspects of MultiBac and its exploits in detail previously [26,27,28]. Therefore, here, we focus only on key features relevant to mammalian multigene delivery, ultimately for CRISPR-mediated gene editing and genome engineering.

**Figure 2:**
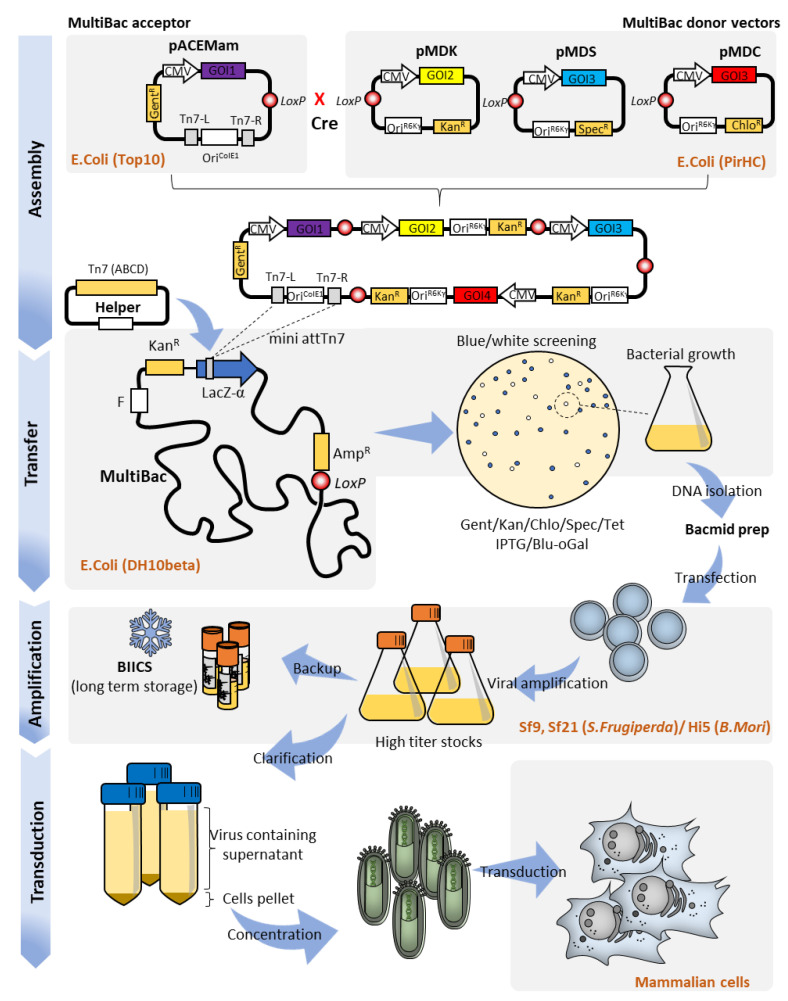
Schematic workflow of MultiBacMam, from construct assembly to mammalian cell transduction for simultaneous delivery and expression of four fluorescently labelled proteins. (Assembly) In this example, four different genes of interests (GOIs: H2B iRFP720, EYFP tubulin, mTFP1 actin, and mito-mCherry) are cloned into pACEMam1, pMDK, pMDC, and pMDS, respectively. Through in vitro controlled Cre-mediated recombination, the 4 plasmids are assembled into a unique vector; (Transfer) The all-in-one vector can now be transformed into DH10beta MultiBac cells which contain the MultiBac genome, outfitted with the Bac-to-Bac locus, and helper plasmid expressing the Tn7 transposase proteins. White bacterial colonies are expanded and cultured to extract the recombinant bacmid DNA; (Amplification) The bacmid DNA is transfected into insect cells (Sf21, Sf9, or Hi5) for the initial stage of virus production. After a few days, the supernatant, containing low titer viral concentration, is used to inoculate fresh cells to obtain high viral titers and the virus containing supernatant is harvested; (Transduction) The virus containing supernatant can be readily used for mammalian cell transduction, or further concentrated by ultracentrifugation to achieve higher transduction efficiencies.

MultiBac relies on in vitro controlled Cre-mediated recombination, a collection of bacterial antibiotic resistance markers, and a conditional replicon for efficient assembly of multigene transfer plasmid which can be readily shuttled into a custom-engineered baculovirus genome, primarily by Tn7 transposition (Figure 2). Following standard procedures, the resulting composite bacmid is extracted and used to transfect insect cells for producing large quantities of the virus. Within insect cells, the recombinant bacmid behaves as a naked DNA virus, and triggers viral amplification without the need for helper or packaging vectors in contrast to other viral systems. The budded virions, which are constantly released into the medium, are typically amplified for one to two passages up to titers as high as 1 × 10^8^–1 × 10^9^ plaque forming units per ml (pfu/mL) (Figure 2).

Next, the virus containing supernatant is cleared by low-speed centrifugation and filtration to remove insect cells and debris and, if needed, can be further concentrated by high-speed centrifugation to obtain higher transduction efficiencies. In analogy to BacMam, a ”MultiBacMam” transduction of mammalian cells is typically achieved between 4 and 6 h of exposure to the virions, while maximal transgene expression levels are observed between 24 and 72 h post transduction (Figure 2). The MultiBac system features a second Cre-*LoxP* entry site distal to the Tn7 attachment site which can be functionalized, for example, with fluorescent proteins (e.g., EYFP, mCherry, etc.) to gauge viral titer during amplification steps. Tropism, which in the viral context typically describes the viral means of entry or host range, can also be modulated by exploiting this site, by introducing genes encoding for envelope proteins from completely unrelated viral species. This process, known as pseudotyping, results in the production of chimeric enveloped baculovirus which can then enter cells and even organisms other than its original insect host.

Using this approach, simultaneous delivery of up to five different transgenes in a variety of mammalian cell lines was achieved with high efficiency, including in cells recalcitrant to plasmid transfection and primary cells [19] (Figure 2). Multiple fluorescently labelled proteins, reporters, and cell-fate driving transcription factors have all been efficiently delivered to mammalian cells for diverse applications ranging from in vivo cell imaging to trans-differentiation [19]. Little to no cytotoxic effect was observed [25]. The latency of the transgene expressions and their persistence in the host cells were transient and largely dependent on initial viral titers and cellular proliferation rates, with the vectors being diluted over time, reminiscent of plasmid transfection [19]. This feature which is commonly considered to be a handicap in traditional gene therapy approaches, can be, in fact, extremely valuable for CRISPR-based approaches, in which Cas9 and sgRNAs expressions need to be precisely controlled and, at some point, disposed of to avoid undesired gene editing outcomes. 

In trend-setting experiments, the MultiBac-based approach was utilized to efficiently deliver, on a single vector, all components required for precise gene editing in mammalian cells (Cas9, sgRNA, and HDR donor), enabling C-terminal tagging of the *HMGA1* gene locus with eGFP in cultured mammalian cells as a proof-of-concept [19,20]. This result is already remarkable when compared with other delivery systems which typically require, for the same editing strategy, co-transduction with two or more viral species.

## 3. Homology-Directed Repair (HDR), Base Editing, and Search-and-Replace Approaches

CRISPR/Cas9 technology enables highly efficient generation of DSBs at desired genome loci with a programmable DNA nuclease guided by a single guide RNA (sgRNA). The sgRNA is a chimeric fusion of CRISPR-associated RNA (crRNA) and trans-activator CRISPR-associated RNA (tracrRNA) [1], and its spacer sequence (e.g., the portion of the sgRNA complimentary to its genomic target) can be designed to match the desired genomic target (protospacer), providing that the latter is followed by a short protospacer adjacent motif (or PAM). The PAM requirement typically depends on the Cas9 variant being used (e.g., NGG for *Steptococcus pyogenes* Cas9 or NNGRRN for *Staphylococcus aureus* Cas9 [8]) and constrains the genomic area of intervention. To expand the universe of the “editable” genome, different Cas9 variants, or other CRISPR systems such as Cas12a [7], CasX [29], or engineered enzymes with altered PAM specificity [8] can be implemented. Online platforms for CRISPR experimental designs can easily design sgRNA candidates which are scored based on their sequence composition, PAM, and number of potential off-targets also implementing scores obtained from high-throughput validation studies where available [30].

Upon successful delivery, the Cas9/sgRNA complex binds to its target region and produces a blunt double stranded break at 3–4 bases upstream of the PAM sequence. Again, different Cas enzymes can produce slightly different DSB outcomes which vary for blunt/staggered ends or relative position to the PAM. For instance, Cas12a cleaves the DNA at 18 bp distance from its -NTT PAM, leaving a five nucleotides staggered DSB [7].

**Figure 3:**
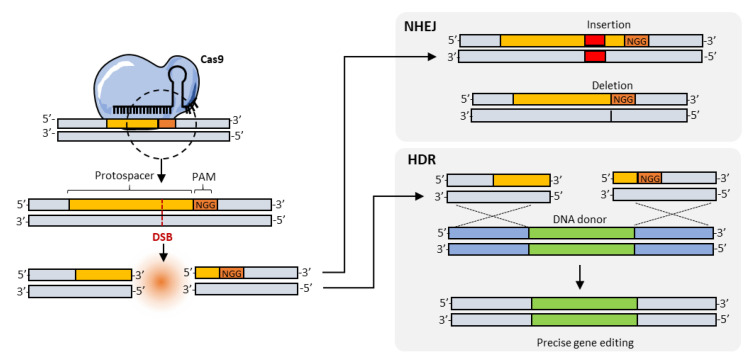
Schematic representation of Cas9/sgRNA-induced double stranded break and subsequent gene editing outcomes. Cas9/sgRNA cleaves the target sequence 3–4 bp before the PAM, leaving a blunt double stranded DNA break which can be repaired through the non-homologous end joining (NHEJ) pathway by introducing small indels or through the homology-directed repair (HDR) pathway which, in the presence of a suitable DNA donor template with left and right homology arms, can faithfully introduce a new DNA fragment in place.

Once the DSB has been produced, endogenous DNA repair pathway components are recruited to repair the DNA damage and influence the gene editing outcome. The non-homologous end joining (NHEJ) pathway is the most efficient DNA repair mechanisms in most cell types and it is constitutively active at all cell cycle stages [9]. Although the NHEJ pathway is referred to as an “error-prone” DNA repair pathway, most of the Cas9-induced DSBs are presumably repaired seamlessly [31], restoring protospacer and PAM sequence creating a loop sequence of DSB induction and repair cycles. Eventually, the NHEJ pathway introduces mutations (usually small insertions and deletions), which alter the protospacer sequence enough to block Cas9-mediate cleavage (Figure 3). Indels can be useful for introducing frameshift mutations and efficiently interrupting gene functions ex vivo but, due to their unpredictability, this approach is not likely to be translated into any in vivo therapy.

Alternatively, DSB can also be repaired through homology-directed repair (HDR) DNA repair pathway. Unlike the NHEJ pathway, the HDR pathway takes advantage of a DNA template which, in virtue of homology arms surrounding a novel DNA fragment, faithfully introduces the new information within the host genome (Figure 3). HDR activity, however, is typically low and cell cycle regulated; mainly confined to the late S and G2 phases [32].

Several strategies have been applied to enhance CRISPR-HDR efficiency ranging from DNA repair pathway modulation to Cas9 variants engineering. NHEJ inhibiting strategies, using small molecule inhibitors of ligase IV such as SCR7, have been shown to nearly double the efficiency of the HDR pathway [33]. Similarly, p53 inhibition to down-tune DNA damage response [34] or expression of engineered ubiquitin inhibitors of 53BP1 [35] (a mediator of end-resection pathway) managed to slightly increase HDR efficiency.

Albeit extremely interesting, it is worth reminding that modulation of the HDR and NHEJ pathways may not be advantageous in vivo, because modulating vital DNA damage response pathways could result in the production of undesired and potentially deleterious long-lasting side effects such as de novo mutations or genomic aberrations. Another interesting and less invasive approach to increase HDR-mediated integration is taking advantage of Cas9 variants engineered to be more stable during cell cycle stages in which the HDR pathway is naturally more active. One such approach utilizes a Cas9 C-terminally fused with a fragment from geminin proteins (derived from the FUCCI cell cycle reporter system [36]) which favors Cas9 accumulation in S/G2 and nearly doubles gene editing efficiency [37]. Similarly, ABT and nocodazole, two inhibitors of microtubule polymerization, have been used to reversibly arrest cell cycle in G2/M phases to enhance CRISPR/HDR-mediated gene editing in human pluripotent stem cells (hPSCs) [38].

Remarkably, higher HDR efficiencies can be achieved by using optimized nonviral delivery systems even in the absence of further Cas9 engineering or HDR/NHEJ ratio manipulation. Roth and colleagues implemented an optimized nonviral delivery electroporation system of Cas9/sgRNAs RNPs complexes and single-stranded oligonucleotide donors (ssODN) templates in cultured cells and obtained up to 60% correct integration and simultaneous knock-in in up to three different loci [10], albeit at the expense of mild cytotoxicity (30% cell death) and relatively small insert size (700 bp excluding the short homology arms). This approach has been successfully used to edit and engineer TCR receptors in primary human T-cells [10] which are notoriously recalcitrant to transfection and poorly transduced by most viral vectors. The positive impact of this delivery technology on the increased HDR-mediated knock-in efficiency could probably be attributed to the high number of ssODN delivered per cell but its translation in vivo would be extremely challenging.

The efficiency of CRISPR/Cas9 (and Cas variants) to induce DSBs is a double-edged sword. Although overexpression of Cas9 results in higher editing efficiency, it can cause off-target cleavage and, in the presence of a DNA donor, can lead to random (and undesired) genomic integration. Furthermore, considering the diploid nature of the human genome, even in the presence of successful knock-in or gene replacement, most cells contain one corrected allele and the other wild-type or, worse, are randomly mutated with unpredictable effect on the protein production outcome. Edited cells ex vivo can be easily screened for the absence of off-targets, undesired integrations, or homozygous knock-in events, but the situation is totally different in vivo.

While the off-target problem can be partially dealt with by using more specific Cas9 variants such as EvoCas9 [39] or Cas9-HF [40] and by improving the sgRNA design, the on-target repair outcomes are again often resolved with random indels.

Given the inefficiency of HDR-based editing, combined with high off-target rates and the unpredictability of the unwanted on-target repair events, alternative gene editing strategies based on catalytically inactive programmable nucleases have been developed. Mutations in the RuvC 1 (D10A) and HNH (H840A) domains of Cas9, respectively, abolish complimentary and non-complimentary DNA strand cuts [1]. Cas9 D10A and Cas9 H840A are still able to bind the sgRNA and their DNA target, but only produce the nicking of one DNA strand, whereas a combination of both mutations completely abolishes the enzymatic activity, resulting in a catalytically dead Cas9 (dCas9). When fused to cytosine deaminase, dCas9 can work as a base editor (BE), resulting in single base changes within the target sequence producing C•G to T•A conversion [41]. Additionally, the repertoire of base editing possibilities has been expanded by the development of adenine base editors (ABEs), in which Cas9(D10A) is coupled to an engineered adenine deaminase resulting in A•T to G•C conversions [42]. Remarkably, these approaches are more efficient than HDR-based editing as they do not rely on endogenous DNA repair pathways and produce virtually no by-products because, in the absence of successful editing, less than 0.1% indels are produced [42].

A more recent “search and replace” approach, named prime editing (PE), relies on Cas9 H840A fused to the Moloney murine leukemia virus reverse transcriptase (MMLV-RT). Combined with an engineered prime-editing sgRNA (PegRNA), the PE binds its target DNA and nicks one strand, whereas the MMLV-RT domain creates a short cDNA using the nicked strand as primer and the PegRNA as template. Then, the newly hybridized cDNA is processed by flap excision-ligation repair and stably incorporated into the genome. The authors showed that PE can efficiently perform all 12 single base substitutions and insert triplets with varying efficiencies and indel rates, mostly dictated by the PegRNA design and the target locus [17]. Amongst the potential drawbacks of both BE and PE, are the very limited size for the gene editing intervention (one to three base pairs for BE and −80, +44 base pairs for PE) and the toxic effects of the associated enzymes at the cellular level. However, given their superior efficiency (up to 75% editing with BE and 50% with ABE) [41,42] and their enhanced safety as compared with DSB-editing approaches, base editors and search and replace approaches are more promising than HDR-based strategies for single base corrections or codon substitutions with direct implications for a variety of disease relevant genes mutations.

While no DNA template is required for DNA editors, larger Cas9 variants are required for both BE (200 kDa, 5.4 kb) and PE (240 kDa, 6.3 kb) as compared with standard SaCas9 (130 kDa, 3.2 kb), Cas12a (143 kDa, 3.8 kb) or SpCas9 (163 kDa, 4.2 kb).

**Figure 4:**
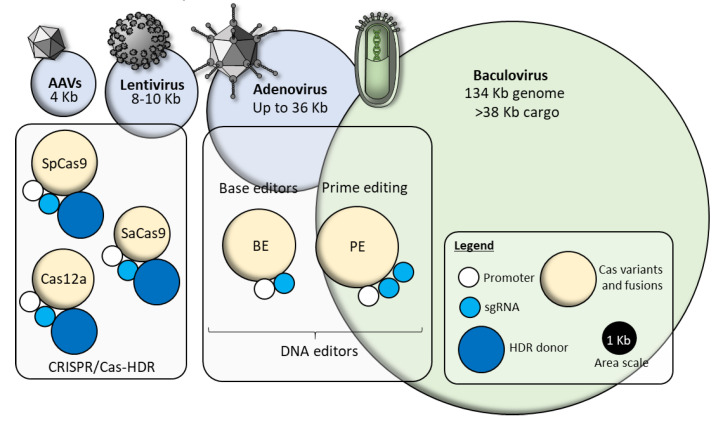
Schematic representation of the size of molecular components required for precise gene editing. Area of the circles approximately represents their DNA size in kb. A black circle (1 kb) is showed in the legend as reference. (Top) Size comparison of standard viral delivery vectors and baculovirus. The area represents the maximum cargo size; (Bottom) For the HDR donor, an insert of 1 kb flanked by two 500 bp homology arms is represented. sgRNA modules area is calculated including the promoter. Yellow circles represent Cas variants or dCas9/nCas9 fusions with base modifies (BE) or MMLVRT (prime editing, PE). A typical constitutive promoter (e.g., cytomegalovirus (CMV) promoter, white circle) is represented in all the constructs, driving the expression of the Cas variant.

Although there is no shortage of available strategies for precise gene editing, a careful evaluation of efficiency versus safety must be undertaken in each experimental approach, together with the PAM availability and with the final gene editing outcome desired (zygosity, toxicity of the undesired editing events, etc.). Foreseeably, more approaches will be developed in the future to reduce off-targets and undesired gene editing outcomes, while increasing efficiency and specificity. Nevertheless, all the available (and presumably future) precise gene editing strategies, rely on the simultaneous delivery of two or more molecular components, often of considerable sizes which challenges the cargo capacity of most viral vectors. Nonviral deliver could possibly cope with the increasing size and number of the required components, but at the expenses of cell-type optimization, manufacturing costs, and being limited to ex vivo approaches in controlled environments for precise gene editing [10,11,13,43]. Baculovirus delivery vectors, on the contrary, can host an impressive cargo size and can efficiently delivery all the examined gene editing approaches as all-in-one toolkits, maximizing the chance of precise genomic intervention (Figure 4). In combination with the MultiBac DNA assembly technology, baculovirus delivery vectors constitute an excellent modular platform for assembly and efficient delivery of different CRISPR toolkits to mammalian cells.

## 4. MultiBac and Clustered Regularly Interspaced Palindromic Repeats (CRISPR)

HDR-based CRISPR gene editing has already been implemented in BVs, leading to a substantial gain in delivery efficiency of all-in-one vectors with modest improvements in gene editing efficiencies [19,20,44]. Although multiple strategies could be implemented to increase HDR efficiency, in the context of BVs, they would take a heavy toll on vector engineering time and represent an unnecessary DNA cargo burden which should be avoided for future in vivo applications.

Rather than forcing target cells to commit to HDR repair, the NHEJ pathway can be exploited to produce targeted insertions in the absence of homology arms, by simply redesigning the donor DNA template. As previously discussed, the NHEJ pathway is mistakenly referred to as an “error-prone” DNA repair pathway. Indeed, Maresca and colleagues uncovered, in 2013, the previously underestimated precision of the NHEJ repair pathway and, in combination with transcription activator-like effector nucleases (TALEN) or zinc finger nucleases (ZFNs), used it to precisely integrate up to 15 kb of DNA donors into mammalian cells through a technique called obligate ligation-gated recombination (ObLiGaRe) [45].

The same holds for CRISPR-based approaches and, by slightly changing the design of the donor DNA, NHEJ-based editing has been shown to efficiently produce knock-ins in dividing and non-dividing cells at base pair precision. Furthermore, this strategy, named homology-independent targeted integration (HITI), does not require homology arms and it works in vivo, producing up to 60% of correct editing [18,46,47,48].

**Figure 5:**
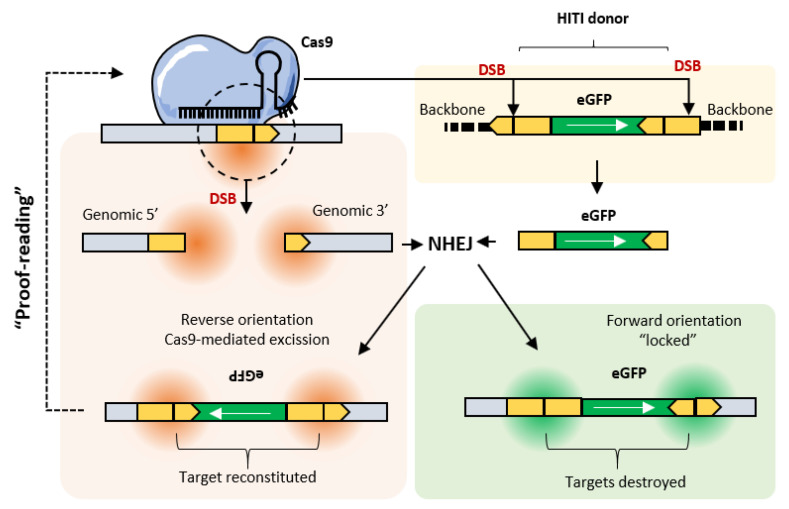
Proposed mechanisms for homology-independent targeted ligation (HITI). Cas9 produces 1 double stranded DNA break (DSB) at the selected genomic locus and excises the HITI donor by cleaving the same target sequence flanking the DNA fragment to be inserted. The excised HITI donor is ligated into the genomic site through the NHEJ pathway. Reverse integrations are corrected by continuous excision/repair cycles in virtue of flanking target sites reconstitution. Forward integrations, containing the desired editing outcome, are “locked” in place and cannot be processed further.

HITI works by excising the donor DNA, exploiting the same Cas9 target site used for the genomic destination locus. Upon cleavage, the donor is released and it is seamlessly ligated to seal the genomic DSB. The orientation of the integrated donor should be random (50% forward and 50% reverse) but Suzuki and colleagues showed that up to 98% of the integrations contained a forward insertion [18,46]. Indeed, thanks to a clever sgRNAs design on the donor, the reverse integration restored both flanking target sides and, if Cas9 and donor molecules were present, the locus was continuously edited through a “proofreading” activity. Consequently, forward integrations, in which the sgRNAs targets were destroyed, were locked in place and immune from further editing (Figure 5).

HITI is a promising editing strategy to overcome the low efficiency of HDR-based editing and extend genomic intervention to non-dividing cells in vivo. The gene editing efficiency could be possibly improved by the generation of all-in-one vectors followed by viral delivery. In this regard, however, HITI poses an additional challenge for standard viral vectors which are routinely packaged in mammalian cells, i.e., how to prevent Cas9 expression and donor excision during the packaging stage?

The self-cleaving nature of an HITI-based viral vector, would prevent the successful generation of an all-in-one vector including Cas9, sgRNA, and donor module within mammalian cells (e.g., HEK293T). In this case, indeed, the vector would be self-cleaved during viral packaging leading to the generation of donor-free vectors capable only of inducing indels or severely impairing viral production and stability. In baculovirus all-in-one vector, however, viral packaging within insect cells would be a strong advantage as mammalian cell promoters are typically not expressed in insect cells. This would ensure that no Cas9 is expressed during viral packaging in Sf21 cells, preserving virions integrity and delivering intact all-in-one vectors.

**Figure 6:**
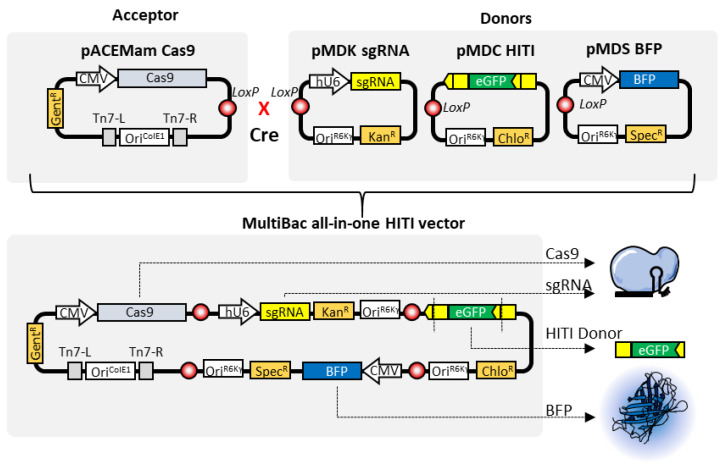
Schematic representation of MultiBac clustered regularly interspaced palindromic repeats (CRISPR) assembly for homology-independent targeted integration. A donor (pACEMam) loaded with Cas9, is fused to sgRNA, HITI template and CMV BFP donors (as a transduction reporter) using CRE-mediated recombination. The resulting all-in-one vector can be readily used to produce baculoviral particles. The modular nature of the assembly system enables easy repurposing for different targets and donors, as well as fluorescent reporters.

In this regard, MultiBac could be repurposed to have a separate function in each one of the donor/acceptor vectors (Cas9, sgRNA, HITI donor, fluorescence reporter, etc.) to ensure maximum flexibility and modular design (Figure 6).

Furthermore, an attractive perspective in the gene editing field is the docking of a large DNA fragment (>10 kb) at a single base position in the genome for exon or whole gene replacement or to incorporate a new piece of genetic information to implement new cellular functions. This could include whole synthetic gene regulatory networks, signaling pathways, or engineered enzymatic cascades. The purposes of large DNA cargo insertions, at precise loci, span from gene therapy to synthetic biology and industrial applications. Large cargo DNA docking, however, is a challenging task for most CRISPR strategies, regardless of the delivery system. Although NHEJ-based approaches have already been used for large cargo docking of up to 15 kb [45], the efficiency in this case has been severely limited by the delivery method. By combining HITI and MultiBac, in contrast, precise docking of large DNA fragments could be readily implemented by repurposing Cre-mediated fusion to expand the donor size of the all-in-one vectors, while maintaining high transduction efficiency.

**Figure 7:**
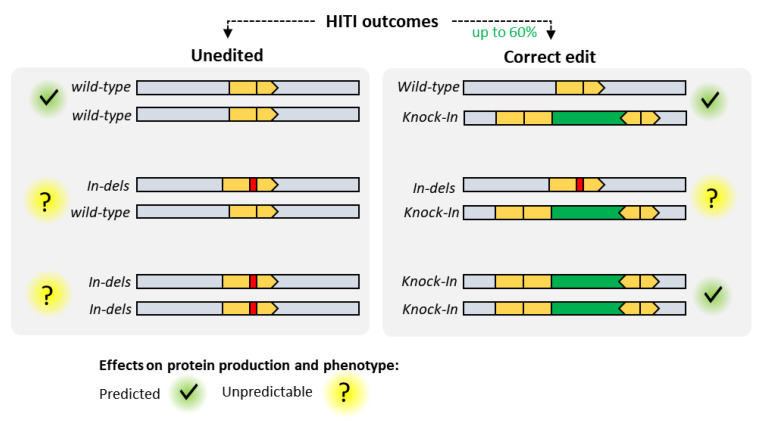
Homology-independent targeted integration and HDR-based approaches relying on active Cas9 produces a complex pattern of gene editing outcomes at the cell population level which must be carefully considered for in vivo applications where clonal isolation and extensive allele screening cannot be achieved.

Although HITI has been successfully used to correct disease-causing genes in vivo [46], despite the high gene editing efficiency, it has to be noted that a consistent proportion of the alleles would still carry on-target indels. HITI intervention generates a complex pattern of repair outcomes and zygosities which must be carefully evaluated for each target before embarking into in vivo experiments because, depending on the target gene, indels could result in aberrant phenotypes (Figure 7). To minimize the side effects linked to DSBs production in vivo, optimized strategies such as intron targeting or whole exon or gene replacement would be advisable, helping to reduce the unpredictability of undesired indels.

Importantly, MultiBac will be equally capable of accommodating and delivering base editors and prime editing CRISPR-based toolkits. As previously discussed, these approaches, which rely on catalytically inactive or impaired Cas variants, are intrinsically safer in virtue of the lack of DSBs. Despite the reduced number of components required for precise editing, base and prime editing require larger engineered Cas effectors, which significantly increase the size of the all-in-one vectors and cannot be efficiently delivered using a single AAV or lentiviral vector. However, this can be easily assembled on baculoviral vectors (Figure 4) and efficiently delivered, preserving the same modularity and strategy depicted in Figure 6. We foresee that base editors and prime editing, delivered through all-in-one MultiBac vectors, should be a game changing technology suitable for in vivo applications in virtue of superior safety, enhanced delivery efficiency, and seamless gene editing outcome with few to no by-products.

## 5. Challenges to Baculovirus Delivery In Vivo

When combined with CRISPR and homology-independent targeted integration, baculovirus expression vectors engineered through MultiBac technology could potentially overcome many of the issues imposed by standard viral vectors. While further studies are required to address safety and efficacy of baculovirus delivery in vivo, key potential challenges have been addressed (summarized in Table 1). Despite wild-type virus life cycle being well characterized, the features that regulate baculoviral transduction in mammalian cells, from entry to innate immune response, have remained elusive. It is well known that BV transduction efficiency, despite being often high, is pronouncedly cell-type dependent and there are certain cell types which are poorly or ineffectively transduced. A key aspect has been addressed by studying the entry route of BVs in mammalian cells. The entry of wild-type AcMNPV into insect and mammalian cells is mediated by binding of the major envelope glycoprotein GP64 to the host cell surface. GP64 is an essential component of budded virions, which is not only involved in cell-to-cell transmission [49] but is also required for viral budding from insect cells [50]. Although the nature of the interactions of GP64 with specific membrane receptors is unclear, efficient entry of budded viruses has been shown to be low-pH and cholesterol dependent [51,52]. Similar to most enveloped viruses, clathrin-dependent endocytosis seems to be the main process involved in baculovirus internalization in mammalian cells, together with macropinocytosis [51,53]. In the endosomes, acidification triggers GP64-mediated membrane fusion, which results in the release of the viral nucleocapsid into the cytoplasm [54,55]. Studies have revealed that capsids are actively transported towards the nuclear compartment via polymerization of actin filaments and enter the nucleus through the nuclear pore complex [56,57]. In addition to rationally designed protein fusions, directed evolution experiments on GP64 have revealed that the E45K/T259A double mutant improved up to eight-fold transduction of human airway epithelia [58]. The SVNs’ transduction and resistance to serum can also be chemically enhanced by treatment with polymers such as polyethylene glycol (PEG) or polyethylenimine (PEI) [59,60,61,62]. However, PEG and PEI coating strategies are not free of risks. For instance, a high degree of PEG coating (PEGylation) significantly reduces baculovirus titer [62]. Anti-PEG immunity [63] must be carefully considered in PEG-baculovirus formulations, and PEI could be subject to similar problems [64]. Additionally, baculovirus high titer stocks have been shown to have a reduced shelf life due to the formation of non-functional aggregates [65]. Given that both PEG and PEI increase the baculovirus molecular weight, further studies are required to address the impact of these coating strategies on the formation of non-functional aggregates.

Although the baculovirus ably transduces a wide range of mammalian cell lines with high efficiency, some cell types seem to be completely refractory to AcMNPV transduction. To overcome this limitation, much effort has been put into engineering the baculoviral envelope. BVs displaying intact or truncated versions of the VSV envelope glycoprotein (VSV-G) are particularly popular [19,66,67]. Because desired peptides or proteins can be easily displayed on the viral membrane by direct fusion to full-length or shortened versions of GP64, various protein motifs have also been explored to achieve cell-specific targeting [78,79,80,81]. Pseudotyping of glycoproteins from naturally occurring viruses can be included, granting even broader or cell-type specific tropism. In addition to VSV-G [68], baculoviruses displaying other molecules have been successfully engineered. These include rabies virus glycoprotein (RVG) [69] and glycoproteins from Thogoto or Dhori viruses [71] with cell type dependent improvements on transduction efficiency. Additionally, baculovirus tropism can be altered by introducing cell-specific peptides extensively tested for redirecting AAVs or lentiviral vectors [70].

Accumulating evidence has suggested that baculoviral genomes could be rapidly inactivated and silenced by innate intracellular immune response pathways [76,77]. This phenotype, again, cell type dependent, has been traditionally counteracted by the addition of small molecule inhibitors of histone deacetylases (HDACs) including valproic acid (VPA) or sodium butyrate (NaBu) [74,75]. While these molecules have shown remarkable improvement of transgene expression in most cell lines, HDAC inhibitors were toxic and could not be administered in vivo. The mechanisms of baculoviral transgene silencing in mammalian cells were likely to be mediated by pattern recognizing receptors (PRRs) and their downstream signaling effectors, including stimulator of interferon genes (STING) [76] and cells defective for the immune-response pathway components such as STING, TBK1, IRF3, or IPS-1 have shown higher baculovirus transgene expression [76]. Interestingly, a short hairpin library study identified RIPK1 as a target, the ablation of which did not result in detectable cytotoxicity, while enhancing baculovirus mediated transgene expression [77], suggesting that transient intracellular immune response modulation, ideally by heterologous modalities hardwired into the baculoviral genome, could be a viable strategy to further improve BV mediated transduction.

Baculoviral vectors lack pre-existing immune response in humans [21], however, they have not naturally evolved to escape the innate immune response mediated by the complement cascade system [82]. Early studies showed that BVs were rapidly inactivated by human serum complement and that inhibition of serum complement restored their transduction potential [83]. Towards systemic baculovirus delivery for in vivo applications, successful strategies have been deployed to counteract complement-mediated inactivation through complement shielding pseudotyping. For instance, decay-accelerating factor (DAF) [72] or DAF fused to complement regulatory proteins [73] have been successfully displayed on the viral surface to shield it from serum complement together with a variety of other strategies which have been authoritatively reviewed previously [84].

## 6. From MultiBac to Synthetic Virus-Derived Nanosystems (SVNs)

During viral production, overamplification of recombinant baculovirus is avoided to prevent spontaneous recombination and formation of defective interfering particles (DIPs) [19,85]. Unlike AAVs or lentiviral vectors, baculovirus expression vectors have not been extensively modified and the AcMNPV genome used in all BVs has remained substantially identical. Because BVs have been extensively used and studied to produce recombinant proteins and protein complexes, the functions of most of the 156 genes of wild-type AcMNPV have been characterized or inferred by homology [22]. By studying baculoviral genomes evolutionary conservation, combined with literature-based knowledge, we previously ranked ORFs based on their likeliness to be deleted without affecting viral production [86]. Building on this knowledge, design and testing of synthetic MultiBac genomes (SynBac) [28] should result in vectors with reduced size, improved stability, and better suited for indefinite amplification and faithful gene transfer into mammalian cells.

**Figure 8:**
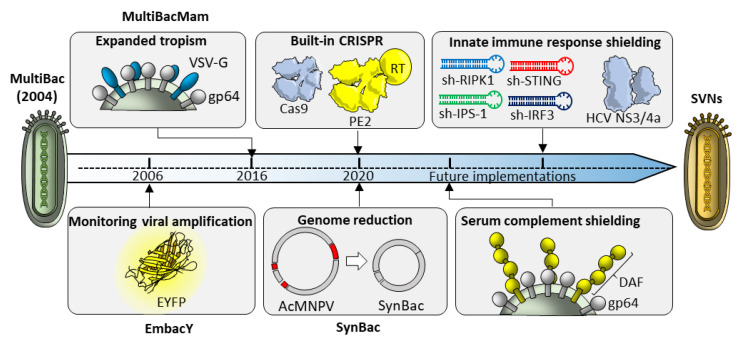
Evolution of baculoviral genomes building up from MultiBac technology. Since its first implementation, MultiBac allowed easy baculoviral genomes and hosting of different Built-in reporter or pseudo-typing components have contributed to enhance virus production (EmbacY) and transduction in mammalian cells (MultiBacMam). In addition to the incorporation of CRISPR components on synthetic and minimized genomes (SynBac), genetically encoded strategies for innate immune response shielding (RNA interference or protease-based), and decay accelerating factor (DAF) pseudo-typing, will enable in vivo systemic administration into living organisms.

In order to tackle current challenges, minimization, design, and rapid re-adaptation of synthetic baculovirus genomes for different target cells/administration routes are crucial features for their successful implementation as in vivo gene delivery vectors. Through extensive and rational baculovirus engineering, it should be possible to gradually move from AcMNPVs to synthetic virus-derived nanosystems (SVNs), while preserving transduction efficiency and high viral production titers and simultaneously implementing molecular strategies to circumvent known roadblocks to BV mediated in vivo delivery. Initial steps in simplifying baculoviral genome engineering have been implemented in the MultiBac system [87]. Customized MultiBac genomes have been developed hosting diverse modules hardwired in the viral backbone. For instance, MultiBac genomes have been successfully functionalized adding fluorescent probes to monitor viral titers [87], pyrolysyl tRNA/tRNA synthetase for genetic code expansion [88], or mammalian glycosylases and others [89] (Figure 8).

The ongoing development of synthetic minimal baculoviral genomes (SynBac) should maximize cargo to backbone capacity and stability, facilitating the implementation of modular functions depending on the final application. Of note, preliminary tests with SynBac1.0, in which a 10 kb fragment (7.6% of the wild-type genome) was ablated, have already resulted in a synthetic virus with improved genome stability over prolonged passaging and high-level protein production [28]. Among the advantages of synthetic minimized genomes is the potential to combine several features into a single, optimized backbone. For instance, in gene editing applications, modules encoding Cas9 or BE/PE2 machinery, could be directly incorporated into the synthetic genome, rather than being provided separately through Tn7 recombination (Figure 8). In the same vein, for in vivo applications, several genetically encoded strategies such as altered pseudotyping [68,71] or serum complement shielding [72] could be combined into SVNs with enhanced tropisms and activity in living organisms. 

The range of interventions to optimize in vivo SVNs’ potential, could also span genetically encoded strategies to tame intracellular pathways which lead to baculovirus inactivation. These could include RNA interference against known mediators of viral inactivation [76,77] or the inclusion of ortholog viral proteases [76] (e.g., NS3/4a protease from Hepatitis C virus) with known immuno-suppressing activities to enhance transgene expression and gene editing efficiency (Figure 8).

## 7. Conclusions and Outlook

Proof-of-concept experiments using MultiBac confirmed that baculoviral vectors have the potential to become a powerful platform to deliver elaborate CRISPR machineries in mammalian cells granting high transduction efficiency and enhanced safety, situated at the boundary between viral and nonviral delivery systems and combining advantages of both. If outfitted with HITI for NHEJ-mediated precise gene editing, baculovirus holds the unique potential of being able to carry all-in-one vectors which are currently impossible to assemble or deliver with alternative viral vectors. Furthermore, in virtue of their extended cargo capacity, BVs, and in particular, the more advanced SVNs, hold the potential to overcome many of the limitations imposed by AAVs or lentiviral vectors, further expanding the range of gene editing intervention to large transgene docking and whole gene replacement.

Despite the absence of pre-existing immunity, baculovirus, in general, is still subject to innate immunity inactivation. Serum complement rapidly inactivates baculoviral particles, whereas, at the cellular level, PRRs recognize and inactivate baculoviruses through different sets of recognition/effector pathways. Additionally, baculoviral glycoprotein (gp64) does not have a wide mammalian cell tropism and it is known that baculoviral genomes cannot be amplified indefinitely due to spontaneous recombination events, increasing the cost of manufacturing high-quality virions, albeit this can conceivably be overcome by engineering towards enhanced stability. Although a whole range of applications is already possible using multigene assembly technologies such as MultiBac, a further step in vector engineering must be undertaken to design BVs which can reliably be used as in vivo delivery vectors. To tackle these challenges, with the aim of generating optimal vectors for gene editing in vivo, a gradual process, started with MultiBac genomes engineering, aims at designing and implementing synthetic viral genomes with reduced DNA burden and complexity, which can be further functionalized with multiple genetically encoded components to meet the needs for industrially manufactured in vivo delivery vectors for a variety of applications. These synthetic virus-derived nanoparticles (SVNs) should ideally combine enhanced stability, selective tropism, and superior activity, with shielding from the innate immune system through ”stealth” envelopes, and the capacity to deploy intracellular immune-suppressing components and modalities.

We anticipate that SVNs could make for an excellent all-in-one CRISPR delivery platform which could be iteratively modified towards large DNA precision docking and bona fide genome engineering, capable of erasing and rewriting large sections of target genomes. Unlike currently available CRISPR delivery systems, SVNs would enable scientists to engineer the delivery vector to meet their needs, rather than adapting the gene editing approach to the delivery system, while offering an unprecedented range of gene editing interventions ex vivo and in vivo in virtue of virtually little to no DNA cargo constraints.

## Figures and Tables

**Table 1:** Known challenges to baculovirus administration in vivo and a summary of strategies to overcome them.

Baculovirus in Vivo Delivery Challenges	Approaches to Overcome Issue
Narrow tropism (e.g., target cells or tissue are not efficiently transduced)	Pseudotyping can be used to change or expand cell tropism: Vescicular stomatitis virus glycoprotein, VSV-G [19,66,67,68]; Rabies virus glyprotein RVG [69]; Cell-type specific peptides [70]; Thogoto and Dori virus envelope proteins [71]; Gp64 mutants [58].
Serum complement-mediated inactivation Baculovirus is inactivated by human serum complement-cascade	Pseudotyping with complement shielding factors can enhance viral stability in the bloodstream: Decay accelerating factor (DAF) [72]; Chimeric DAF fused to complement regulatory factors [73]. Chemical modifications have been reported to enhance serum resistance: Polyethylene glycol (PEG) coating [62]; Polyethylenimine (PEI) coating [60,61].
Intracellular immune response inactivation Baculovirus efficiently reaches target cells but is rapidly inactivated and silenced by intracellular immune response pathways.	Histone deacetylases (HDACs) inhibitors can be used ex vivo to counteract silencing. Due to broad spectra and high toxicity they cannot be used systemically: •Valproic acid (VPA) or sodium butyrate (NaBu) [74,75]. Genetically encoded intracellular immune suppression strategies increase transgene expression: Ablation of STING, TBK1, IRF3, or IPS-1 [76]; RNA interference of RIPK1 [77]; Viral proteins with intracellular immuno-suppressive activity (NS3/4a protease from Hepatitis C virus) [76].
